# The *GJA8* allele encoding CX50I247M is a rare polymorphism, not a cataract-causing mutation

**Published:** 2009-09-14

**Authors:** Jochen Graw, Werner Schmidt, Peter J. Minogue, Jessica Rodriguez, Jun-Jie Tong, Norman Klopp, Thomas Illig, Lisa Ebihara, Viviana M. Berthoud, Eric C. Beyer

**Affiliations:** 1 Helmholtz Center Munich - German Research Center for Environmental Health, Institute of Developmental Genetics, D-85764 Neuherberg, Germany; 2 Center of Ophthalmology, Universities of Gießen and Marburg, D-35385 Gießen, Germany; 3 Department of Pediatrics, Section of Hematology/Oncology, University of Chicago, Chicago, IL; 4 Department of Physiology and Biophysics, Rosalind Franklin School of Medicine and Science, North Chicago, IL; 5 Helmholtz Center Munich - German Research Center for Environmental Health, Institute of Epidemiology, D-85764 Neuherberg, Germany

## Abstract

**Purpose:**

The aim of this study was the genetic, cellular, and physiological characterization of a connexin50 (CX50) variant identified in a child with congenital cataracts.

**Methods:**

Lens material from surgery was collected and used for cDNA production. Genomic DNA was prepared from blood obtained from the proband and her parents. PCR amplified DNA fragments were sequenced and characterized by restriction digestion. Connexin protein distribution was studied by immunofluorescence in transiently transfected HeLa cells. Formation of functional channels was assessed by two-microelectrode voltage-clamp in cRNA-injected *Xenopus* oocytes.

**Results:**

Ophthalmologic examination showed that the proband suffered from bilateral white, diffuse cataracts, but the parents were free of lens opacities. Direct sequencing of the PCR product produced from lens cDNA showed that the proband was heterozygous for a G>T transition at position 741 of the *GJA8* gene, encoding the exchange of methionine for isoleucine at position 247 of CX50 (CX50I247M). The mutation was confirmed in the genomic DNA, but it was also present in the unaffected mother. When expressed in HeLa cells, both wild type CX50 and CX50I247M formed gap junction plaques. Both CX50 and CX50I247M induced gap junctional currents in pairs of *Xenopus* oocytes.

**Conclusions:**

Although the CX50I247M substitution has previously been suggested to cause cataracts, our genetic, cellular, and electrophysiological data suggest that this allele more likely represents a rare silent, polymorphic variant.

## Introduction

Congenital cataracts are responsible for approximately 10% of childhood blindness worldwide. They are clinically and genetically heterogeneous. More than 30 loci have been linked to the cataract phenotype, and at least 17 cataract-associated genes have been characterized including those encoding crystallins, transcription factors, cytoskeletal proteins, and membrane proteins (reviewed in [1,2]). Among these, mutations in the *GJA3* and *GJA8* genes (that encode the lens gap junction proteins, CX46 and CX50) have been shown to underlie congenital cataracts, which most often exhibit dominant inheritance [3]. The CX46 and CX50 mutants that have been characterized do not support intercellular communication when expressed by themselves (and some of these mutants act as dominant negative inhibitors of wild type connexin function) supporting the hypothesis that decreased gap junctional intercellular communication contributes to cataract formation.

A nucleotide transition in *GJA8* (T741G) that co-segregated with the cataract phenotype was previously identified in a Russian family [4]. The cellular and functional properties of this mutation have not been characterized. In the current study, we have examined the cellular and physiological consequences of the encoded amino acid substitution (CX50I247M) and report the identification of the same substitution in members of another family in which the transition does not co-segregate with the cataract phenotype.

## Methods

The study adopted the tenets of the Declaration of Helsinki. Family members gave informed consent after explanation of the study design and goals and their roles. The study was approved by the Institutional Ethical Committee of the University of Gießen, Germany.

Clinical details regarding the health histories of family members were recorded at the Center of Ophthalmology at the University of Gießen (Germany). A senior pediatric ophthalmologist (W.S.) performed surgery on the proband. Lens material from cataract surgery was frozen immediately on dry ice and kept at -80 °C for a few days only [5]. RNA samples from lenses were reverse transcribed to cDNA using the Ready-to-Go kit (GE Health Care, Freiburg, Germany). Using this cDNA as template, PCR was performed to amplify crystallin, alpha A (*CRYAA*); crystallin, alpha B (*CRYAB*); crystallin, beta A4 (*CRYBA4*); crystallin, beta B1 (*CRYBB1*); crystallin, beta B2 (*CRYBB1*); crystallins, gamma A-D (*CRYGA-D*); crystallin, gamma C (*CRYGC*); *GJA8*; ferritin, light polypeptide (*FTL*); lens intrinsic membrane protein 2, (*LIM2*); and major intrinsic protein of lens fiber (*MIP/AQP0*). The selection of these genes was based upon the frequency of mutations in these genes leading to congenital cataracts.

The parents were examined for the presence of lens opacities with a slit-lamp (Zeiss, Oberkochen, Germany). Blood samples (5-10 ml) were collected from the proband and her parents, and they were used to isolate genomic DNA [6]. The conditions for PCR reactions performed using genomic DNA as template have been described previously [7-9]. Primers were obtained from Utz Linzner (Helmholtz Center Munich, Institutes of Experimental Genetics and of Pathology, Munich, Germany) or from commercial sources (Invitrogen, Karlsruhe, Germany, MWG, Vaterstetten, Germany or Sigma Genosys, Steinheim, Germany). Sequencing was performed commercially (Sequiserve, Vaterstetten, Germany or GATC Biotech, Konstanz, Germany). The presence of the mutant allele in the PCR fragments was confirmed by LweI digestion.

The Cooperative Health Research in the Augsburg Region (KORA) Survey 2000 (S3) which studied a population based sample of 4,261 subjects aged 25–74 years during the years 1999–2001 [10] was used as a population-based control. 179 randomly chosen individuals without cataracts from this cohort were analyzed for the putative *GJA8* mutation.

The human wild type CX50 coding sequence was subcloned into pSP64TII [11] and pcDNA3.1/Hygro(+) (Invitrogen Life Technologies, Carlsbad, CA) [12]. The mutant allele (CX50I247M) was generated in these expression plasmids using a PCR-based site-directed mutagenesis strategy [13,14]. The coding regions of the PCR products were sequenced to confirm the fidelity of the amplification reaction.

HeLa cells were grown on 4-well chamber slides and transiently transfected with CX50 or CX50I247M (in pcDNA3.1/Hygro) [14]. Forty-eight hours after transfection, cells were fixed, and immunofluorescence was performed using affinity purified rabbit polyclonal anti-CX50 antibodies [15].

Connexin DNAs (in pSP64TII) were transcribed and capped in vitro, and cRNAs were injected into defolliculated *Xenopus* oocytes that had been injected with an oligonucleotide antisense to the endogenous *Xenopus* CX38 [16]. Oocytes were paired and studied after 14-18 h by double two-microelectrode voltage-clamp recording to allow determination of junctional conductance (g_j_) [17]. Animals were maintained and treated in accordance with NIH/PHS policies on humane care and use of laboratory animals.

## Results

**Figure 1:**
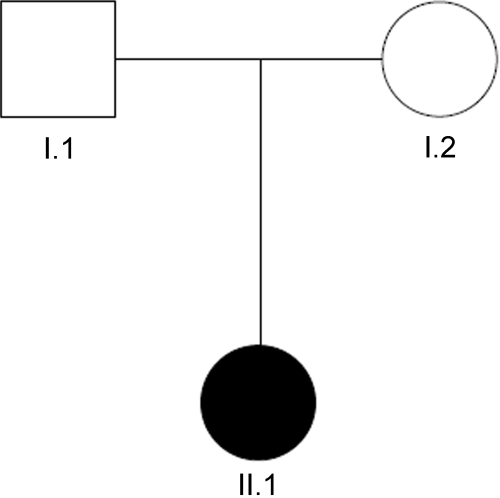
Pedigree of family B. The pedigree shows a classical trio of healthy parents (I.1 and I.2) and an affected child (II.1).

The proband, LB, suffered from bilateral, diffuse white lens opacities. She underwent cataract surgery shortly after birth. Both parents were healthy; slit lamp examination showed no evidence of lens opacities (Figure 1).

**Figure 2:**
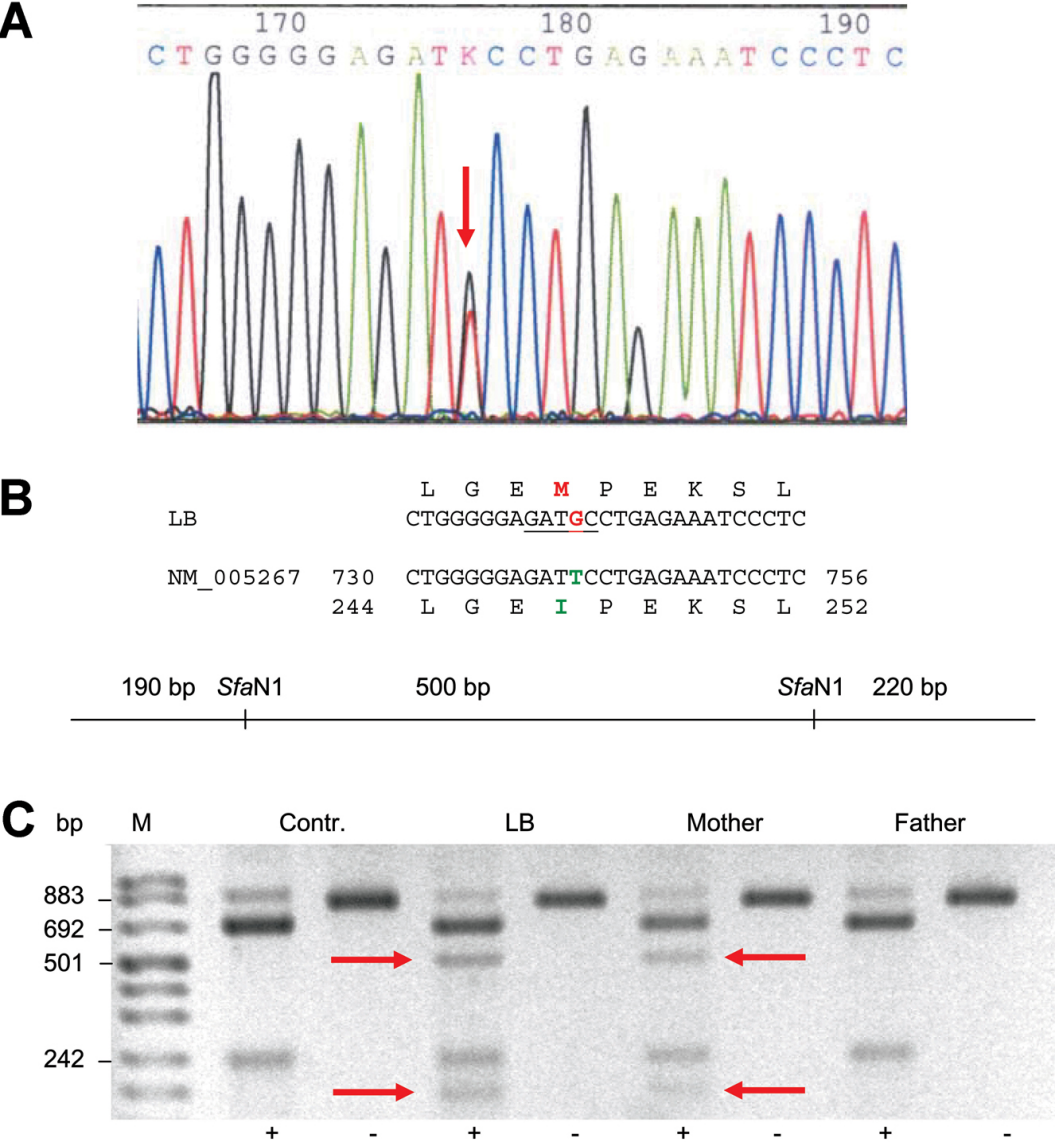
Molecular analysis of proband LB and her parents. **A**: Sequence analysis of *GJA8* cDNA indicates a T/G heterozygosity at position 741 (red arrow) for the proband (II.1). **B**: The T→G exchange at position 741 leads to an amino acid exchange from Ile to Met at position 247 (I247M) and creates an SfaN/LweI restriction site in the mutated sequence. **C**: Restriction digest using LweI in the members of the family demonstrates its presence also in the unaffected mother (red arrows).

Using a functional candidate approach, we checked several genes including *GJA8* for sequence alterations. We identified a T→G exchange in *GJA8* cDNA at position 741 (Figure 2A). This substitution changes the amino acid codon at position 247 from isoleucine to methionine (CX50I247M). It also creates a new SfaN/LweI restriction site in the mutated sequence (Figure 2B). Using LweI digestion of the PCR fragments obtained from genomic DNA, we observed the same transition in the unaffected mother (Figure 2C, arrows). Sequencing of genomic DNA from both parents confirmed that the mother was heterozygous like the daughter, and the father was wild type. None of the other genes analyzed (*CRYAA*, *CRYAB*, *CRYBA4*, *CRYBB1*, *CRYBB2*, *CRYGA-D*, *CRYGS*, *FTL*, *LIM2*, and *AQP0*) showed alterations.

We also used LweI digestion of genomic DNA to test for the presence of the CX50I247M allele in 179 controls obtained from a population-based study (KORA). Since no additional LweI restriction site was observed in these samples, the frequency of the CX50I247M allele must be less than 0.3%.

**Figure 3:**
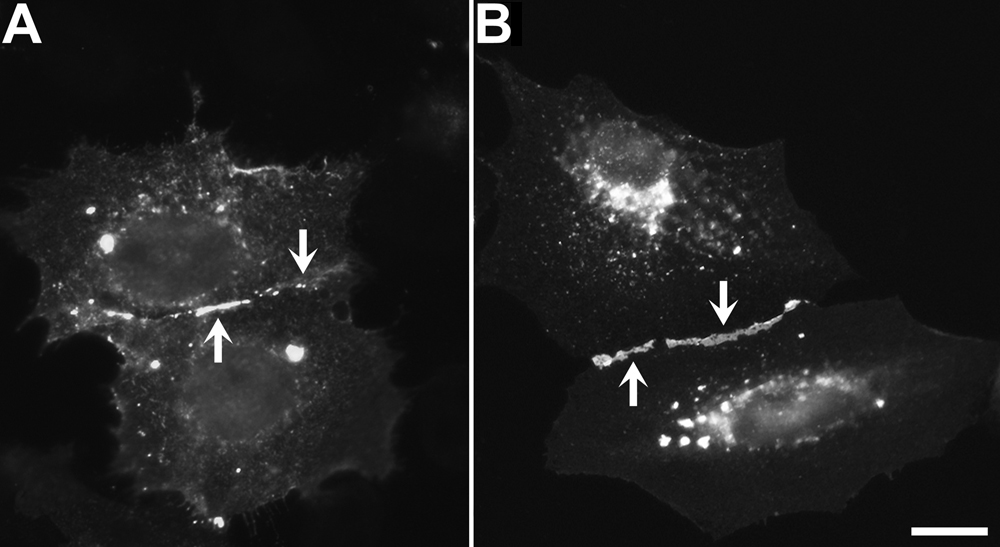
Immunodetection of wild type and mutant CX50 in transfected HeLa cells. **A**, **B**: HeLa cells transfected with CX50 or CX50I247M were fixed 48 h after transfection and subjected to immunofluorescence using anti-CX50 antibodies. The distribution of CX50 immunoreactivity appeared similar in both groups of cells; cells expressing either CX50 or CX50I247M showed a significant number of gap junctional plaques (arrows). The scale bar represents 13 μm in **A** and 17 μm in **B**.

The capacity of CX50I247M to form gap junctions was assessed by immunofluorescence microscopy of HeLa cells transfected with wild type CX50 or CX50I247M. Similar to wild type CX50, CX50I247M localized at appositional membranes, where it formed gap junction plaques, and in the perinuclear region, probably the Golgi compartment (Figure 3A,B).

**Figure 4:**
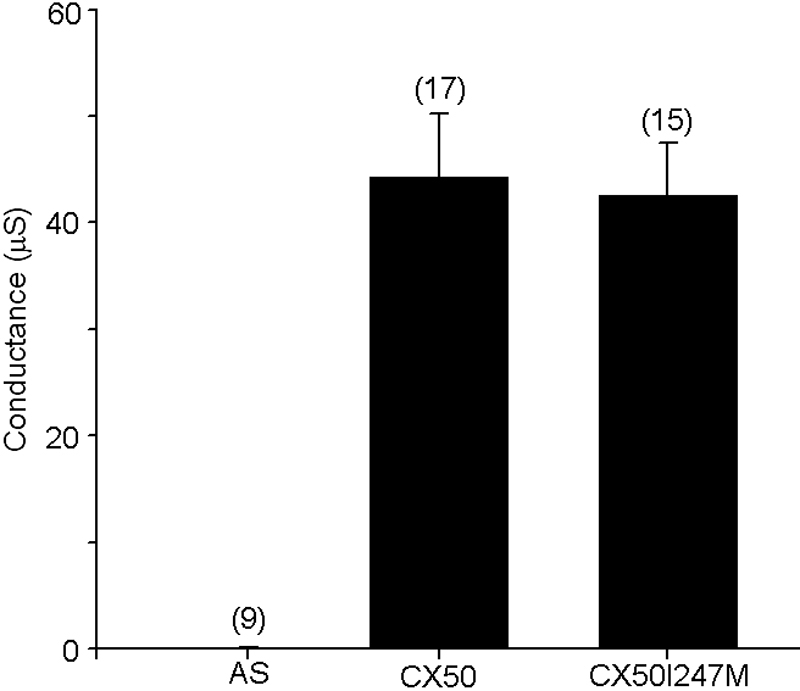
Gap junctional conductances induced by wild type or mutant CX50. Graph shows a summary plot of steady-state gap junctional conductances in pairs of *Xenopus* oocytes injected with cRNAs encoding wild type CX50 or CX50I247M or injected with no connexin cRNA (AS). Results are presented as mean ± .E.M. Numbers in parentheses indicate the number of oocyte pairs studied in each case.

The ability of CX50I247M to form functional gap junctional channels was characterized by two-electrode voltage-clamp in *Xenopus* oocyte pairs. Pairs of oocytes injected with CX50I247M cRNA developed gap junctional conductances with mean values that were not significantly different from those determined in oocyte pairs injected with wild type CX50 cRNA (Figure 4). Pairs of control oocytes injected with no connexin cRNA showed no coupling.

## Discussion

In this study, we demonstrated a heterozygous mutation in *GJA8* of a child with severe congenital cataracts (LB). The mutated sequence encodes CX50I247M, a CX50 variant in which the isoleucine at position 247 (within the cytoplasmic COOH-terminus) is replaced by methione. CX50I247M formed gap junction plaques and supported intercellular communication similarly to wild type CX50.

Very few of the identified cataract-associated connexin mutations lie in the COOH-terminus. Indeed, removal of the COOH-terminus (139-150 amino acids) of CX50 causes only modest effects on voltage-dependent gap junction channel gating [18,19]. Similar to the effects caused by truncation of ovine Cx50 [20], removal of the COOH-terminus of human CX50 results in a decrease in sensitivity to intracellular pH (pH_i_) [18]. Truncation of mouse CX50 also appeared to cause a decrease in sensitivity to pH_i_ as evidenced by the delay in the decrease in junctional conductance induced by 100% CO_2_ perfusion and the slower recovery of gap junctional conductance following washout [19]. Truncated human and mouse CX50 both show decreased junctional conductance [18,19]. Thus, this region may be important for regulation of CX50 channel function, but it is dispensable for channel activity per se. Two of the mutations in lens connexin genes linked to hereditary cataracts that affect the COOH-terminus cause frame shifts [5,21]. In the Cx46 mutant, CX46fs380 (that contains a frame shift at codon 380), the new protein sequence caused by the frame shift contains a retention/retrieval signal that leads to loss of function [22] and localization of the mutant connexin in the cytoplasm [13].

The CX50 variant, CX50I247M, was previously reported in three members of a three generation Russian family, and it co-segregated with a zonular pulverulent cataract trait [4]. However, this mutation did not co-segregate with the disease in our study; it was also present in the healthy mother of our proband. (Indeed, the genetic alteration responsible for the cataract in our patient has not been identified.)

The segregation of the CX50I247M allele with cataract in the Russian family remains puzzling [4], because it seems to be a rare allele with a frequency of less than 0.3%. A plausible explanation for the contradicting findings between the previous study and ours is the possibility of a close linkage to another gene, which is really causative for these cataracts. If this hypothesis were true, there should be another cataract-related gene close to the CX50-encoding gene *GJA8*. Referring to the database of Mendelian hereditary disorders (OMIM), a few further cataract loci are reported on human chromosome 1; however, two of them have already been attributed to *GJA8*. Another one is the gene encoding glyceronephosphate O-acyltransferase (*GNPAT*) for which a syndromic cataract would be expected rather than an isolated one as we have reported. Therefore, one might speculate that there is another yet unidentified cataract-associated gene in this region. Examination of the ENSEMBL database for this region reveals a considerable number of genes that have not yet been annotated including some genes for non-coding RNAs.

Our cellular and functional studies support the conclusion that CX50I247M is an inconsequential variant. Taken together, our results strongly suggest that CX50I247M represents a rare polymorphic site rather than a causative mutation.
